# Effect of sealer coating on hardness and water sorption data of soft denture lining materials

**DOI:** 10.1016/j.dib.2021.107083

**Published:** 2021-04-24

**Authors:** Ivana Ivana, Ismet Danial Nasution, Darwin Yunus Nasution, Ariyani

**Affiliations:** a Postgraduate Program in Prosthodontic, Faculty of Dentistry, Universitas Sumatera Utara, Medan 20155, Indonesia; b Department of Prosthodontic, Faculty of Dentistry, Universitas Sumatera Utara, Medan 20155, Indonesia; c Department of Chemistry, Faculty of Mathematics and Natural Sciences, Universitas Sumatera Utara, Medan 20155, Indonesia; d Department of Prosthodontic, Faculty of Dentistry, Universitas Sumatera Utara, Medan 20155, Indonesia

**Keywords:** Shore hardness, Water sorption, Soft denture lining, Sealer coating

## Abstract

Soft denture lining (SDL) are acrylic or silicone based materials that can be cured with heat or auto polymerization process, which is commonly used in removable prosthodontics to reline the intaglio (interior) surface of the denture. Loss of softness due to aging process is caused by loss of plasticizer and other soluble component. However, water sorption causes changes in structure and increased materials hardness, thus need frequent replacement of the materials. To extend the durability of the SDL, sealer coating (SC) was used. In this data, sealant coating acts as mechanical barrier to prevent water sorption and solubility of the chemical component to preserved material hardness. This article provides the data of sealant coating effect on hardness and water sorption of acrylic-based and silicone-based SDL materials was arranged in 4 test group: pristine and coated acrylic SDL and pristine silicone SDL and coated silicone SDL. Shore Hardness test was carried out using a Shore A Durometer and water sorption data were presented. Accordingly, the data was statistically analyzed for comparison using independent T-test for shore A hardness and water sorption.

## Specifications Table

**Table:** 

Subject	Biomaterials
Specific subject area	Dental materials
Type of data	Figure Table
How data were acquired	Hardness test using shore A durometer (Kori KR14 A, Japan). Water sorption analysis was obtained according to the American Dental Association (ADA) experimental procedure.
Data format	Raw Analyzed
Parameters for data collection	There are two different soft denture lining (SDL) namely acrylic and silicon of pristine and sealant coated materials. The shore A hardness and water sorption tests were determined.
Description of data collection	The experimental data were carried out and analysed in Department of Prosthodontic, Faculty of Dentistry, Universitas Sumatera Utara, Medan 20155, Indonesia
Data accessibility	All data herein and supplementary files are available within this article.

## Value of the Data

• The data of hardness and water sorption of acrylic and silicone based soft denture lining (SDL) without and with coating are essential for distribution and absorption of masticatory loads to minimize trauma for the supporting tissue underneath the denture.
• The data can be beneficial for both researchers and dental clinician for utilizing the sealant coating on acrylic and silicone based SDL materials.
• The data is useful for further research on other alternatives of various sealant coating materials.
• The data can be used to compared with other suitable coating for SDL materials.

## 1 Data Description

**Fig. 1:**
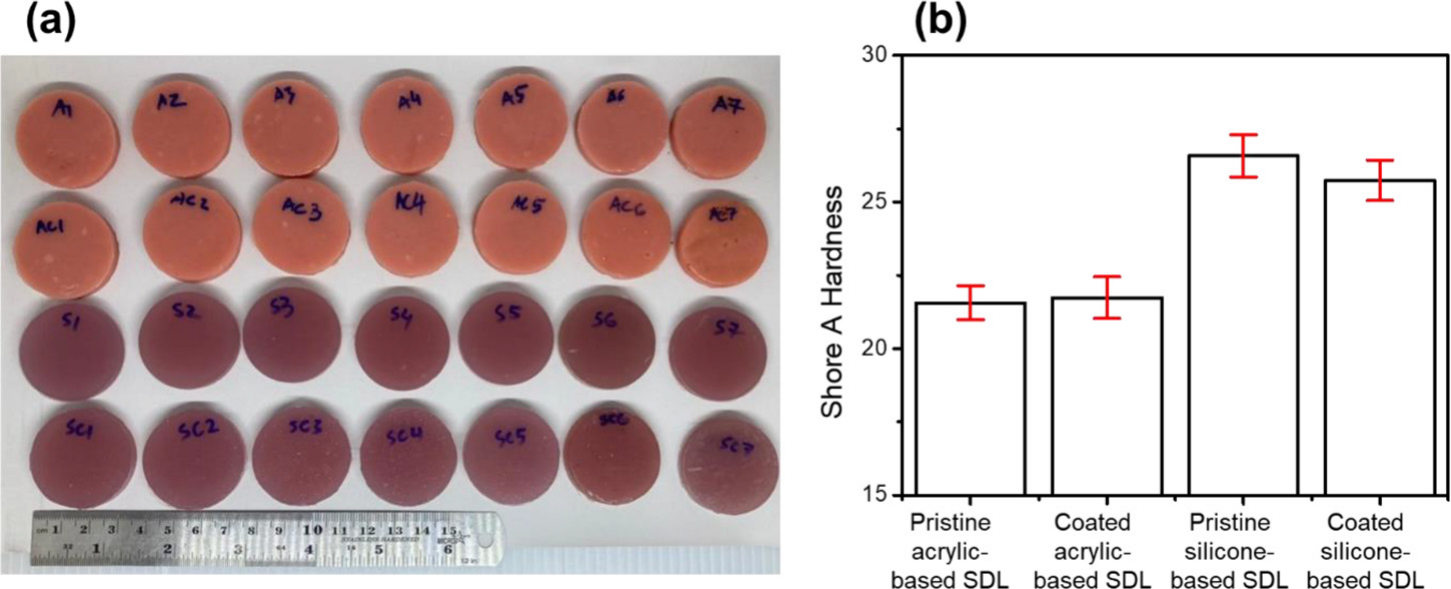
(a) photograph image and (b) shore A hardness of pristine and coated of both acrylic and silicon-based SDL materials.

The dataset of mean and standard deviation for shore hardness testing was presented in Fig. 1 and the data was summarized in Table 1. Independent T-test was performed to determine the effect of sealant coating on the hardness of soft denture lining (SDL) materials. T-test between pristine and coated acrylic-based SDL was p=0.696 (p > 0.05), which can be refer to supplementary materials. In addition, the T-test between pristine and silicone coated-based SDL group shows p value of 0.077 (p > 0.05), thus there is no significant effect of SC on both silicone and acrylic coated SDL materials shore hardness in comparison with pristine SDL group.

**Table 1:** Summary of Shore A hardness of pristine and coated acrylic and silicone-based SDL materials.

No. of Sample	Pristine acrylic-based SDL	Coated acrylic-based SDL	Pristine silicone-based SDL	Coated silicone-based SDL
1	21.50	21.00	27.40	25.10
2	21.40	21.30	25.60	26.20
3	20.80	21.40	26.10	26.70
4	22.40	21.00	27.00	25.20
5	21.70	22.50	26.80	25.50
6	23.30	22.50	27.80	25.70
7	22.50	22.70	25.30	26.00
**Average**	**21.94 ± 0.84**	**21.77 ± 0.76**	**26.57 ± 0.93**	**25.77 ± 0.57**

**Fig. 2:**
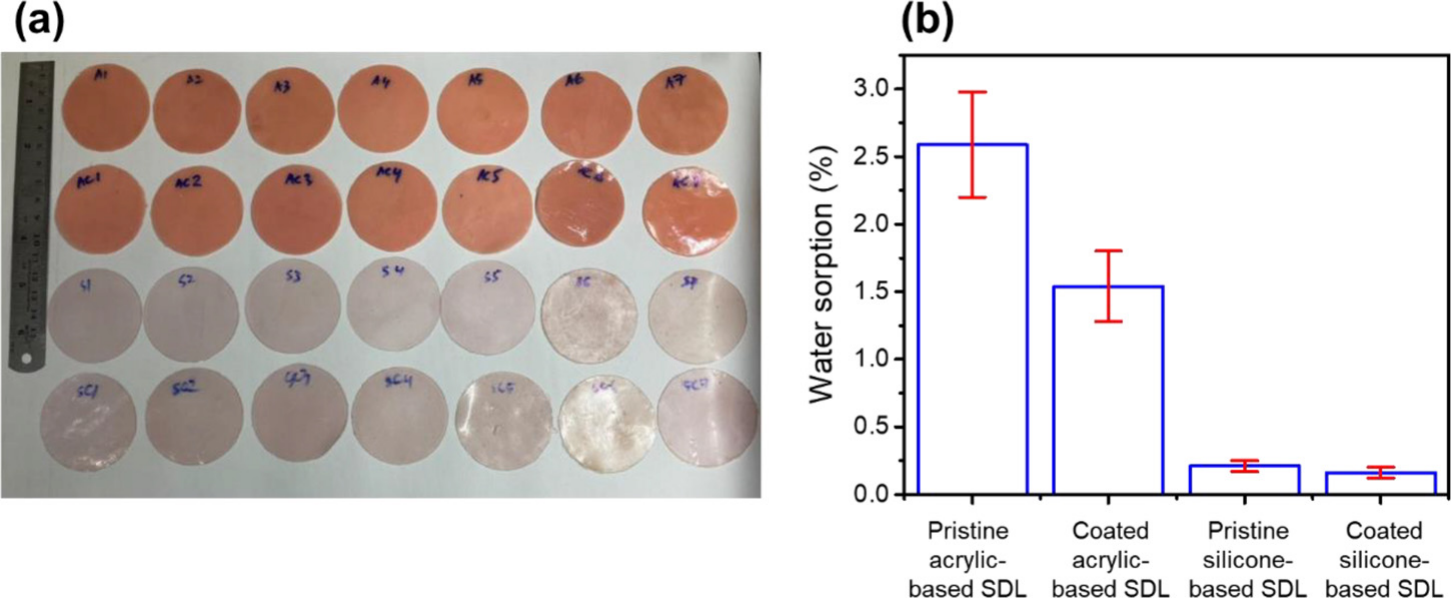
(a) Photograph image, (b) water sorption (%) of pristine and coated of both acrylic and silicone based SDL materials.

The percentage of water sorption calculated can be seen in Fig. 2 and Table 2. Independent T-test between coated and uncoated acrylic-based SDL is p = 0.001 (p < 0.05), where there is a significant effect of SC on water sorption of acrylic-based SDL. T-test between coated silicone-based SDL group and pristine silicone-based SDL group also shows a significant effect of sealant coating on the water sorption between coated and pristine silicone-based SDL groups (p = 0.043). The Shore A Hardness value was after immersion the sample for 24 h in distilled water at 37 °C, therefore for the soft types was within the range of 25-50 shore hardness (silicone) and for extra soft types was below 25 shore hardness units (acrylic) in accordance to ISO 10139-2: 2016 for soft lining materials.

**Table 2:** Summary of water sorption data from each sample of pristine and coated of both acrylic and silicone-based SDL materials.

Number of sample	Pristine acrylic-based SDL	Coated acrylic-based SDL	Pristine silicone-based SDL	Coated silicone-based SDL
1	2.75	1.39	0.20	0.10
2	2.10	1.77	0.15	0.14
3	2.53	1.50	0.21	0.13
4	2.95	1.84	0.28	0.21
5	2.03	1.17	0.18	0.15
6	2.98	1.34	0.24	0.19
7	2.78	1.78	0.21	0.20
**Average**	**2.59 ± 0.39**	**1.54 ± 0.26**	**0.21 ± 0.04**	**0.16 ± 0.04**

## 2 Experimental Design, Materials and Methods

In this dataset, we used auto polymerized acrylic-based SDL (DuraBase Soft, Reliance Dental Manufacturing LLC, Illionis, USA) and auto polymerized silicone-based SDL (Mollosil, Detax GmbH, Ettlingen, Germany). The sealant coating was purchased from Lustrol, Detax GmbH, Ettlingen, Germany) auto polymerized silicone-based SDL. Monopoly syrup was made by mixing 1 part of clear auto polymerized polymer powder with 10 part of heat polymerized acrylics monomer in a water bath at 55°C and stirred until it thickens for 8 min, which resulting in 20 µm using surface profilometer (Dektak 150). The total samples in this study were 56 samples consisting of 28 samples for hardness testing and 28 samples for water sorption testing. The samples were then divided into 4 groups, each of group consisted of 7 samples: pristine auto polymerized acrylic-based SDL, coated acrylic-based SDL, pristine auto polymerized silicone-based SDL and coated silicone-based SDL. Samples were prepared by pouring the above materials into a disc shaped-stainless steel mold with a diameter of 35 mm and a thickness of 6 mm for Shore hardness test and disc-shaped stainless steel mold (diameter of 50 mm and thickness 0.5 mm) for water adsorption test [1]. The mold was closed up to 20 min for polymerization processed. After that, the sample was then removed from the mold, meanwhile for the coated group, sealer coating was applied using a thin brush evenly in one direction, then allowed to dry for 5 min. After drying, it was recoated in the same way for three times [2].

Shore hardness test was performed in accordance with the International Organization for Standardization (ISO) standards ISO 10139-2:2016 for long term resilient denture liner. All samples were placed into an incubator at 37 °C for 24 h prior to the measurement. The shore A durometer (Kori KR14 A, Japan) was calibrated according to ASTM D2240 and the hardness test was recorded in Shore units. Each shore unit represents 0.0254 mm deflection by the durometer indenter. The tip of the durometer indenter which is a truncated cone with a length of 2.5 mm and using an 822 g spring, it is placed on the sample slowly until the sample surface and the contact surface of the durometer form one plane and the reading of the hardness value was recorded according to previous procedure [3]. Furthermore, five points for each disc samples were carried out (Fig. 1a). The loading points was distributed on the surface and roughly 12 mm from the edge of the sample, therefore five points was obtained and the mean value of shore hardness was calculated. For water sorption, the samples were dried in a vacuum desiccator containing silica gel for 24 h until a constant weight. After that, the samples were weighed on a digital analytical balance (Sartorius, Japan) for recording the weight before immersion (*w_1_*). Subsequently, the sample was immersed in 20 mL of distilled water and stored for 7 days in incubator at 37 °C. The samples were removed from the water and filtered, then the samples were left in ambient for 5 min and weighed (*w_2_*). The samples were dried in the desiccator until constant weight were achieved (*w_3_*). The percentage of water adsorption is calculated using the following equation [4]:

$$Water\;sorption\;\left(\%\right)=\frac{W_{2}-W_{3}}{W_{1}}\times 100\%$$

Mean and standard deviation were calculated. The data were calculated using the independent T test with a significance level of p < 0.05 to determine the effect of SC on the shore hardness and water sorption of auto polymerized acrylic-based SDL and silicone-based SDL.

## CRediT Author Statement

**Ivana:** Conceptualization, Investigation, Data curation, Writing - original draft preparation; **Ismet Danial Nasution:** Investigation, Validation, Writing - reviewing and Editing; **Darwin Yunus Nasution:** Supervision, Writing - Reviewing and Editing; **Ariyani:** Writing - Original draft preparation.

## Declaration of Competing Interest

The authors declare that they have no known competing financial interests or personal relationships which have or could be perceived to have influenced the work reported in this article.

## Appendix Supplementary materials

Image, application 1
