# Supplementary material for: Effect of sealer coating on hardness and water sorption data of soft denture lining materials
Source: Data Brief. 2021 Apr 24;36:107083. doi: 10.1016/j.dib.2021.107083 (PMC8122148; doi:10.1016/j.dib.2021.107083)
Supplement: Supplementary file 1 [file mmc1.docx]

**Supplementary materials**

| **Descriptives** | | | | | |
| --- | --- | --- | --- | --- | --- |
|  | SDL | | | Statistic | Std. Error |
| Water sorption (%) | Pristine acrylic | Mean | | 2.588600 | .1461790 |
|  |  | 95% Confidence Interval for Mean | Lower Bound | 2.230913 |  |
|  |  |  | Upper Bound | 2.946287 |  |
|  |  | 5% Trimmed Mean | | 2.597939 |  |
|  |  | Median | | 2.745500 |  |
|  |  | Variance | | .150 |  |
|  |  | Std. Deviation | | .3867533 |  |
|  |  | Minimum | | 2.0294 |  |
|  |  | Maximum | | 2.9797 |  |
|  |  | Range | | .9503 |  |
|  |  | Interquartile Range | | .8489 |  |
|  |  | Skewness | | -.690 | .794 |
|  |  | Kurtosis | | -1.309 | 1.587 |
|  | Coated acrylic | Mean | | 1.540400 | .0973120 |
|  |  | 95% Confidence Interval for Mean | Lower Bound | 1.302286 |  |
|  |  |  | Upper Bound | 1.778514 |  |
|  |  | 5% Trimmed Mean | | 1.544572 |  |
|  |  | Median | | 1.497800 |  |
|  |  | Variance | | .066 |  |
|  |  | Std. Deviation | | .2574632 |  |
|  |  | Minimum | | 1.1698 |  |
|  |  | Maximum | | 1.8359 |  |
|  |  | Range | | .6661 |  |
|  |  | Interquartile Range | | .4395 |  |
|  |  | Skewness | | -.149 | .794 |
|  |  | Kurtosis | | -1.732 | 1.587 |
|  | Pristine silicone | Mean | | .210871 | .0159157 |
|  |  | 95% Confidence Interval for Mean | Lower Bound | .171927 |  |
|  |  |  | Upper Bound | .249816 |  |
|  |  | 5% Trimmed Mean | | .210640 |  |
|  |  | Median | | .209200 |  |
|  |  | Variance | | .002 |  |
|  |  | Std. Deviation | | .0421091 |  |
|  |  | Minimum | | .1474 |  |
|  |  | Maximum | | .2785 |  |
|  |  | Range | | .1311 |  |
|  |  | Interquartile Range | | .0629 |  |
|  |  | Skewness | | .199 | .794 |
|  |  | Kurtosis | | .410 | 1.587 |
|  | Coated silicon | Mean | | .161614 | .0148194 |
|  |  | 95% Confidence Interval for Mean | Lower Bound | .125352 |  |
|  |  |  | Upper Bound | .197876 |  |
|  |  | 5% Trimmed Mean | | .162271 |  |
|  |  | Median | | .154700 |  |
|  |  | Variance | | .002 |  |
|  |  | Std. Deviation | | .0392086 |  |
|  |  | Minimum | | .1048 |  |
|  |  | Maximum | | .2066 |  |
|  |  | Range | | .1018 |  |
|  |  | Interquartile Range | | .0728 |  |
|  |  | Skewness | | -.128 | .794 |
|  |  | Kurtosis | | -1.576 | 1.587 |
| Shore A hardness | Pristine acrylic | Mean | | 21.9429 | .31687 |
|  |  | 95% Confidence Interval for Mean | Lower Bound | 21.1675 |  |
|  |  |  | Upper Bound | 22.7182 |  |
|  |  | 5% Trimmed Mean | | 21.9310 |  |
|  |  | Median | | 21.7000 |  |
|  |  | Variance | | .703 |  |
|  |  | Std. Deviation | | .83837 |  |
|  |  | Minimum | | 20.80 |  |
|  |  | Maximum | | 23.30 |  |
|  |  | Range | | 2.50 |  |
|  |  | Interquartile Range | | 1.10 |  |
|  |  | Skewness | | .402 | .794 |
|  |  | Kurtosis | | -.330 | 1.587 |
|  | Coated acrylic | Mean | | 21.7714 | .28761 |
|  |  | 95% Confidence Interval for Mean | Lower Bound | 21.0677 |  |
|  |  |  | Upper Bound | 22.4752 |  |
|  |  | 5% Trimmed Mean | | 21.7627 |  |
|  |  | Median | | 21.4000 |  |
|  |  | Variance | | .579 |  |
|  |  | Std. Deviation | | .76095 |  |
|  |  | Minimum | | 21.00 |  |
|  |  | Maximum | | 22.70 |  |
|  |  | Range | | 1.70 |  |
|  |  | Interquartile Range | | 1.50 |  |
|  |  | Skewness | | .265 | .794 |
|  |  | Kurtosis | | -2.500 | 1.587 |
|  | Pristine silicone | Mean | | 26.5714 | .35235 |
|  |  | 95% Confidence Interval for Mean | Lower Bound | 25.7093 |  |
|  |  |  | Upper Bound | 27.4336 |  |
|  |  | 5% Trimmed Mean | | 26.5738 |  |
|  |  | Median | | 26.8000 |  |
|  |  | Variance | | .869 |  |
|  |  | Std. Deviation | | .93223 |  |
|  |  | Minimum | | 25.30 |  |
|  |  | Maximum | | 27.80 |  |
|  |  | Range | | 2.50 |  |
|  |  | Interquartile Range | | 1.80 |  |
|  |  | Skewness | | -.162 | .794 |
|  |  | Kurtosis | | -1.483 | 1.587 |
|  | Coated silicone | Mean | | 25.7714 | .21571 |
|  |  | 95% Confidence Interval for Mean | Lower Bound | 25.2436 |  |
|  |  |  | Upper Bound | 26.2993 |  |
|  |  | 5% Trimmed Mean | | 25.7571 |  |
|  |  | Median | | 25.7000 |  |
|  |  | Variance | | .326 |  |
|  |  | Std. Deviation | | .57071 |  |
|  |  | Minimum | | 25.10 |  |
|  |  | Maximum | | 26.70 |  |
|  |  | Range | | 1.60 |  |
|  |  | Interquartile Range | | 1.00 |  |
|  |  | Skewness | | .479 | .794 |
|  |  | Kurtosis | | -.582 | 1.587 |

| **Tests of Normality** | | | | | | | |
| --- | --- | --- | --- | --- | --- | --- | --- |
|  | SDL | Kolmogorov-Smirnov^a^ | | | Shapiro-Wilk | | |
|  |  | Statistic | df | Sig. | Statistic | df | Sig. |
| Water sorption (%) | Pristine acrylic | .229 | 7 | .200^*^ | .871 | 7 | .190 |
|  | Coated acrylic | .239 | 7 | .200^*^ | .909 | 7 | .391 |
|  | Pristine silicone | .205 | 7 | .200^*^ | .975 | 7 | .934 |
|  | Coated silicone | .189 | 7 | .200^*^ | .925 | 7 | .506 |
| Shore A hardness | Akrilik non coat, non thermo | .185 | 7 | .200^*^ | .966 | 7 | .866 |
|  | Akrilik coat, non thermo | .259 | 7 | .169 | .815 | 7 | .058 |
|  | Silikon non coat, non thermo | .168 | 7 | .200^*^ | .954 | 7 | .765 |
|  | Silikon coat non thermo | .127 | 7 | .200^*^ | .961 | 7 | .829 |
| *. This is a lower bound of the true significance. | | | | | | | |
| a. Lilliefors Significance Correction | | | | | | | |

**T-Test**

| **Group Statistics** | | | | | |
| --- | --- | --- | --- | --- | --- |
|  | SDL | N | Mean | Std. Deviation | Std. Error Mean |
| Kekerasan in SHU | Akrilik non coat, non thermo | 7 | 21.9429 | .83837 | .31687 |
|  | Akrilik coat, non thermo | 7 | 21.7714 | .76095 | .28761 |

| **Independent Samples Test** | | | | | | | | | | |
| --- | --- | --- | --- | --- | --- | --- | --- | --- | --- | --- |
|  | | Levene's Test for Equality of Variances | | t-test for Equality of Means | | | | | | |
|  |  | F | Sig. | t | df | Sig. (2-tailed) | Mean Difference | Std. Error Difference | 95% Confidence Interval of the Difference | |
|  |  |  |  |  |  |  |  |  | Lower | Upper |
| Kekerasan in SHU | Equal variances assumed | .001 | .981 | .401 | 12 | .696 | .17143 | .42794 | -.76096 | 1.10382 |
|  | Equal variances not assumed |  |  | .401 | 11.889 | .696 | .17143 | .42794 | -.76193 | 1.10479 |

| **T-Test**   \| **Group Statistics** \| \| \| \| \| \| \| --- \| --- \| --- \| --- \| --- \| --- \| \|  \| SDL \| N \| Mean \| Std. Deviation \| Std. Error Mean \| \| Shore A hardness \| Pristine silicone \| 7 \| 26.5714 \| .93223 \| .35235 \| \| Coated silicone \| 7 \| 25.7714 \| .57071 \| .21571 \| |
| --- | --- | --- | --- | --- | --- | --- | --- | --- | --- | --- | --- | --- | --- | --- | --- | --- | --- | --- | --- | --- | --- | --- | --- |

| **Independent Samples Test** | | | | | | | | | | |
| --- | --- | --- | --- | --- | --- | --- | --- | --- | --- | --- |
|  | | Levene's Test for Equality of Variances | | t-test for Equality of Means | | | | | | |
|  |  | F | Sig. | t | df | Sig. (2-tailed) | Mean Difference | Std. Error Difference | 95% Confidence Interval of the Difference | |
|  |  |  |  |  |  |  |  |  | Lower | Upper |
| Shore A Hardness | Equal variances assumed | 2.870 | .116 | 1.936 | 12 | .077 | .80000 | .41313 | -.10014 | 1.70014 |
|  | Equal variances not assumed |  |  | 1.936 | 9.944 | .082 | .80000 | .41313 | -.12123 | 1.72123 |

**T-Test**

| **Group Statistics** | | | | | |
| --- | --- | --- | --- | --- | --- |
|  | SDL | N | Mean | Std. Deviation | Std. Error Mean |
| Water sorption (%) | Pristine Acrylic-SDL | 7 | 2.588600 | .3867533 | .1461790 |
|  | Coated Acrylic-SDL | 7 | 1.540400 | .2574632 | .0973120 |

| **Independent Samples Test** | | | | | | | | | | |
| --- | --- | --- | --- | --- | --- | --- | --- | --- | --- | --- |
|  | | Levene's Test for Equality of Variances | | t-test for Equality of Means | | | | | | |
|  |  | F | Sig. | t | df | Sig. (2-tailed) | Mean Difference | Std. Error Difference | 95% Confidence Interval of the Difference | |
|  |  |  |  |  |  |  |  |  | Lower | Upper |
| Water sorption (%) | Equal variances assumed | 1.460 | .250 | 5.969 | 12 | .000 | 1.0482000 | .1756073 | .6655846 | 1.4308154 |
|  | Equal variances not assumed |  |  | 5.969 | 10.445 | .000 | 1.0482000 | .1756073 | .6591704 | 1.4372296 |

**T-Test**

| **Group Statistics** | | | | | |
| --- | --- | --- | --- | --- | --- |
|  | SDL | N | Mean | Std. Deviation | Std. Error Mean |
| Water sorption (%) | Pristine silicone SDL | 7 | .210871 | .0421091 | .0159157 |
|  | Coated silicone SDL | 7 | .161614 | .0392086 | .0148194 |

| **Independent Samples Test** | | | | | | | | | | | | | |
| --- | --- | --- | --- | --- | --- | --- | --- | --- | --- | --- | --- | --- | --- |
|  | | Levene's Test for Equality of Variances | | t-test for Equality of Means | | | | | | | | |  |
|  |  | F | Sig. | t | df | Sig. (2-tailed) | Mean Difference | Std. Error Difference | 95% Confidence Interval of the Difference | | |  |  |
|  |  |  |  |  |  |  |  |  | Lower | Upper |  |  |  |
| Water sorption (%) | Equal variances assumed | .095 | .764 | 2.265 | 12 | .043 | .0492571 | .0217469 | .0018748 | .0966395 |  |  |  |
|  | Equal variances not assumed |  |  | 2.265 | 11.939 | .043 | .0492571 | .0217469 | .0018481 | .0966662 |  |  |  |
